# Generation of the SCN1A epilepsy mutation in hiPS cells using the TALEN technique

**DOI:** 10.1038/srep05404

**Published:** 2014-06-23

**Authors:** Wanjuan Chen, Jingxin Liu, Longmei Zhang, Huijuan Xu, Xiaogang Guo, Sihao Deng, Lipeng Liu, Daiguan Yu, Yonglong Chen, Zhiyuan Li

**Affiliations:** 1 The School of Life Sciences, Anhui University, Hefei 230027; 2 The School of Life Sciences, University of Science and Technology of China, Hefei 230027; 3 Key Laboratory of Regenerative Biology, South China Institute for Stem Cell Biology and Regenerative Medicine, Guangzhou Institutes of Biomedicine and Health, Chinese Academy of Sciences, Guangzhou 510530, China; 4 Xiang-Ya School of Medicine, Central South University; 5 Xiang-Ya Boai Hospital, Changsha 410013, China; 6 These authors contributed equally to this work.

## Abstract

Human induced pluripotent stem cells (iPSC) can be used to understand the pathological mechanisms of human disease. These cells are a promising source for cell-replacement therapy. However, such studies require genetically defined conditions. Such genetic manipulations can be performed using the novel Transcription Activator-Like Effector Nucleases (TALENs), which generate site-specific double-strand DNA breaks (DSBs) with high efficiency and precision. Combining the TALEN and iPSC methods, we developed two iPS cell lines by generating the point mutation A5768G in the SCN1A gene, which encodes the voltage-gated sodium channel Nav1.1 α subunit. The engineered iPSC maintained pluripotency and successfully differentiated into neurons with normal functional characteristics. The two cell lines differ exclusively at the epilepsy-susceptibility variant. The ability to robustly introduce disease-causing point mutations in normal hiPS cell lines can be used to generate a human cell model for studying epileptic mechanisms and for drug screening.

Severe mycological epilepsy of infancy (SMEI, also called Dravet's Syndrome) is a disease with several complicated symptoms including severe, intractable epilepsy and co-morbidities of ataxia and cognitive impairment. SMEI is typically resistant to standard anticonvulsant pharmacotherapy^1^. The genetic etiology of this epilepsy involves mutations in sodium channels; such mutations are frequently observed in the SCN1A gene, which encodes the α1 subunit of the sodium channel NaV1.1. Several types of SCN1A mutations such as nonsense, frame-shift, and missense mutations, located at different sites of the SCN1A gene have been identified in patients with SMEI^2^,^3^. The spectrum of epilepsy syndromes might be caused by mutations' location in the SCN1A gene. Mild impairment of this protein causes a predisposition to febrile seizures; intermediate impairment leads to generalized epilepsy with febrile seizures plus (GEFS+), and severe or complete loss of function leads to SMEI^4^. However, such genotype-phenotype correlations have remained inconclusive until recently. Studies using HEK293 cells expressing human Nav1.1 channels bearing SMEI-associated nonsense and missense mutations revealed that these mutations abrogated the function of the sodium channels and attenuated or eliminated inward sodium currents. The decrease in sodium current might underlie neuronal hyperexcitability and cause epileptic seizures^5^. Studies using animal models revealed that Nav1.1 channels with loss-of-function mutations had severely impaired sodium currents in GABAergic inhibitory inter-neurons. These observations were consistent with the hypothesis that the decrease in sodium current might cause hyperexcitability in SMEI^6^. Furthermore, nonlinear loss of sodium current in Purkinje neurons might reduce their firing rates, causing ataxia and related functional deficits^7^. Further studies are required to understand the molecular pathology of SMEI.

The TALEN technology is a powerful tool for genome engineering, which can be used to cleave unique genomic sequences in living cells. The TALEN system has two components^8^; one component is the Transcription activator-like (TAL) effector, which is a virulence factor in plant pathogenic bacteria of the genus Xanthomonas. The native function of TAL effectors is to subvert host genome regulatory networks after translocation into host cells via the bacterial type III secretion system and to bind effector-specific sequences. The second component is the FokI nuclease, which can efficiently cleave DNA to create targeted DNA double-strand breaks (DSBs) in vivo for genome editing^9^. Because dimeric FokI cleaves DNA, these TAL effector nucleases (TALENs) function in pairs to generate DSBs. These DSBs are repaired by cellular non-homologous end joining (NHEJ) or homologous recombination (HR) pathways, which generate targeted gene disruption including small insertions or deletions (InDel). However, homologous recombination (HR) requires a homologous DNA segment as a template for DNA DSB repair; such homologous sequences can be used for gene insertion or replacement^10^,^11^. Therefore, the TALEN technology provides a robust and rapid designable DNA-targeting platform for the analysis and engineering of biological systems.

Studies on neurodegenerative diseases have been impaired by limited experimental access to disease-affected human nervous system tissue^12^. Human induced pluripotent stem cell (hiPSC) technology which enables the epigenetic reprogramming of human somatic cells into a pluripotent state followed by differentiation into disease-relevant cell types and tissues; this technology provides access to virtually unlimited numbers of patient-specific cells for modeling neurological disorders in vitro. The generation of patient-specific iPSCs carrying disease-relevant genetic alterations represents a significant progress for basic biomedical research in this field^13^,^14^,^15^. In such studies, patient-derived disease-relevant cells are compared with cells from normal individuals. However, such comparisons have important caveats. Neurodegenerative diseases generally have late age of onset, long latency, a slow progression of cellular and pathological changes in vivo^16^, and subtle phenotypes in vitro. Furthermore, it is difficult to ascribe differences between the patient-derived and normal cell lines to the disease-relevant lesion because the two cell lines have different genetic backgrounds. Therefore, distinguishing subtle disease-relevant phonotypic changes from unpredictable background-related variations remains challenging.

To develop a genetically defined experimental system to study SMEI pathology, we developed induced pluripotent stem cells (iPSCs) from the skin fibroblasts of individuals with a normal genotype^17^; these cells were named “Nor”. We then used the TALEN technology to generate the A5768G point mutation in the SCN1A gene of the Nor iPSCs to obtain an artificial patient cell line (AP). Therefore the only difference between the normal (Nor) and artificial patient (AP) cell lines is the disease-causing genetic lesion of interest; these cell lines can be used to elucidate the pathogenesis of epilepsy and to examine therapeutic avenues.

## Results

### TALEN efficiency and donor vector construction

**Figure 1:**
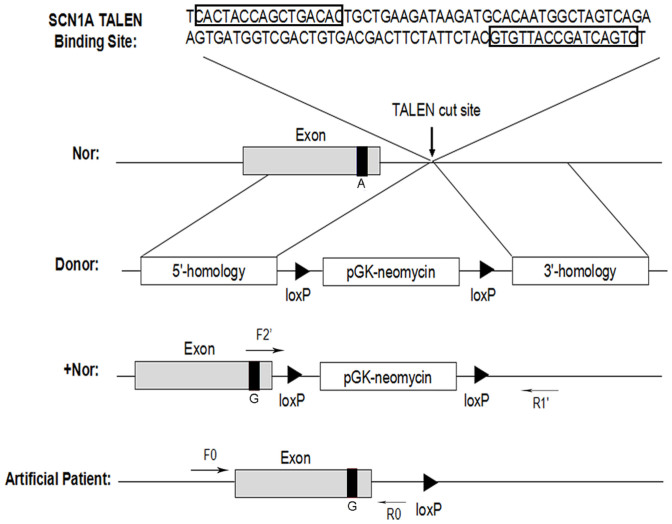
Schematic overview of the targeting strategy for the A5768G mutation The arrows with F_0_/R_0_ and F_2_′/R_1_′ indicate the primers used to test gene targeting. “Nor” indicates the initial normal iPSCs. The donor plasmids contained Neo, the G418-resistance gene for drug screening. “+Nor” indicates the normal iPSCs after gene insertion with the Neo cassette. “Artificial patient” indicates the cells obtained after gene targeting and Cre-mediated excision of the drug cassette.

We sought to use TALENs to introduce the epilepsy-causing mutation A5768G into the endogenous locus of the Nav1.1 α subunit in normal iPS cells (Nor), which were reprogrammed from the skin fibroblasts of a normal individual. We observed that the TALENs designed for *SCN1A* had 7.14% cutting efficiency in the normal iPS cell line without drug selection (Supplemental Table S2). The DNA sequences of the TALENs are shown in Supplemental Figure S1. We used a drug-selection-based strategy to screen the single-cell colonies after electroporation. We constructed the targeting donor vector containing a loxP-flanked PGK-neomycin cassette as well as 5′- and 3′-homology arms that contained approximately 1300 bp of sequence homology flanking the TALEN-targeted site (Supplemental Figure S2). The 5′-homology arm contained the A5768G mutation, which was amplified from the genomic DNA of a SMEI patient. To prevent disruption of target gene expression, the G418-resistance gene (Neo) was inserted in the adjacent intron, approximately 40 bases downstream of the last exon in the SCN1A gene, which encodes the Nav1.1 α subunit (Figure 1).

### Efficient Introduction of disease-related mutations and removal of the neomycin-resistance cassette

**Figure 2:**
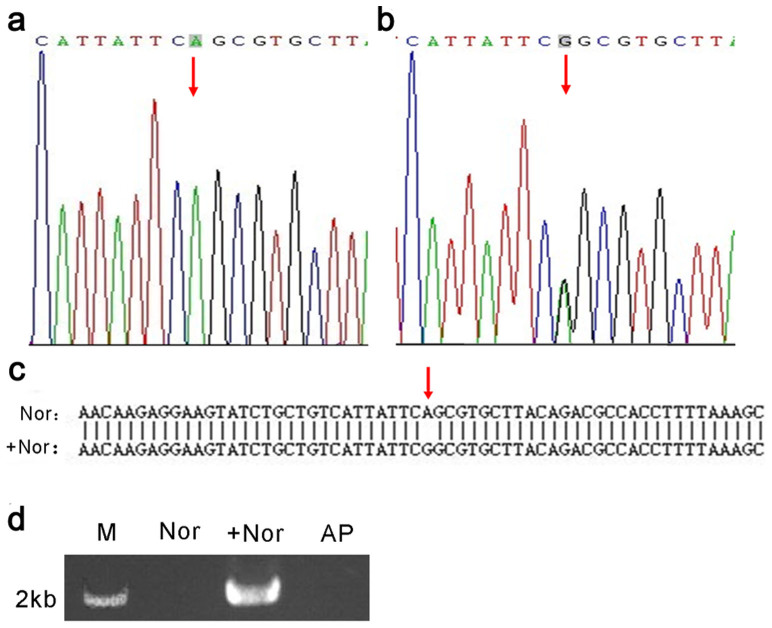
Efficient Introduction of disease-related mutations and removal of the neomycin-resistance cassette Sequencing analyses of the genomic SCN1A locus of normal iPSCs (Nor) and the gene-targeted cell line (+Nor), respectively, are shown in (a) and (b). The base highlighted with a shadow is the disease-related mutation, indicated by red arrows. BLAST analysis of “Nor” and “+Nor” is shown in (c). The red arrow indicates the introduced mutation. (d) DNA gel electrophoresis of the Neo PCR products obtained using the primers R1′/F2′ on DNA from the normal cell line (Nor), the gene-targeted cell line (+Nor) and the “artificial patient” cell line (AP) after removal of the Neo cassette. The full-length gel is shown in Supplemental Figure S4.

To generate mutations in normal iPS cells (Nor), the targeting donor containing the A5768G mutation and the 2 TALEN pairs were electroporated into the iPSC lines. Upon cleavage by TALENs and subsequent homologous recombination with the donor template, the epilepsy-causing A5768G mutation in the donor should be introduced into the endogenous locus of the cell line. We obtained 103 single-cell clones after electroporation and 23 clones after G418 selection. To identify the correctly targeted clones, we designed two pairs of PCR primers: one pair (F_0_/R_0_) was used to amplify the fragment containing the disease-related mutation, and the other pair (R_1_′/F_2_′) was used to amplify the drug-selection cassette. Using the first primer pair (F_0_/R_0_), we always obtained a 1100 bp product irrespective of whether the sequence contained the mutation. The presence of the mutation was then examined by sequencing (Figure 2a/2b/2c). Using the second primer pair (R_1_′/F_2_′), the correctly targeted genome (+Nor) would yield a 2000 bp amplicon, which contains the inserted Neo gene (Figure 2d). And the result of sequencing is shown in Supplemental Figure S3. Further analysis by genomic DNA PCR followed by sequencing revealed that 1 out of 23 G418-resistant clones contained the epilepsy-relevant mutation at the targeted genomic locus. After targeting, the selection cassette (Neo) in the cell lines (+Nor) was excised by introducing a Cre recombinase-expressing plasmid to prevent undesired effects of the residual drug-resistance cassette in the genome. To confirm that the cassette had been excised, we extracted the genomic DNA of single-cell clones and analyzed the DNA by PCR using the primers R_1_′/F_2_′ followed by gel electrophoresis (Figure 2d). This analysis revealed that the drug-resistance cassette was excised in 2 of 91 clones. We named this cell line the “artificial patient” (AP) cell line.

### Characterization of the Nor iPSCs after gene manipulation

**Figure 3:**
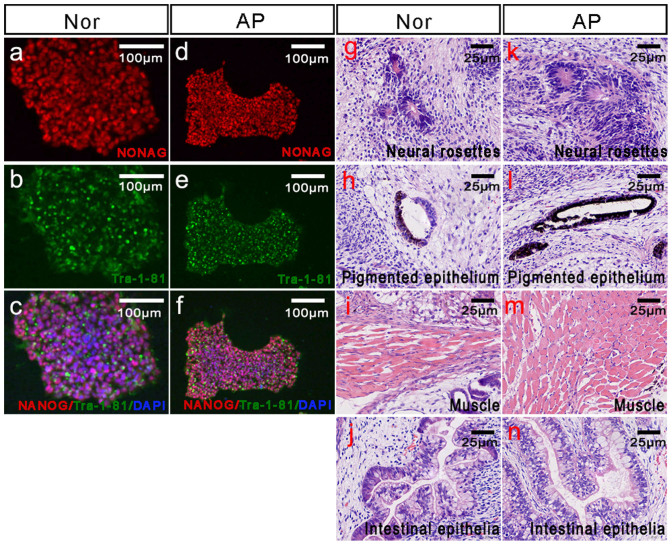
Characterization of Nor iPSCs after gene manipulation Immunofluorescence staining of the “Nor” iPS cells (a/b/c) and the “AP” iPS cells (d/e/f) revealed expression of the pluripotency-specific markers Nanog (red) and TRA-1-81 (green) in vitro. Hematoxylin and eosin staining of teratoma sections of NOD-SCID mice were generated from the “Nor” (g/h/i/j) and “AP” cell lines (k/l/m/n).

First, we examined whether the artificial patient iPSCs were pluripotent. Immunofluorescent staining (Figure 3a/b/c/d/e/f) revealed expression of the pluripotency-specific protein markers Nanog and TRA-1-81 in both the Nor and AP iPSCs. Teratomas were generated by injecting the “Nor” and “AP” iPSCs into immunodeficient mice; HE staining (Figure 3g/h/i/j/k/l/m/n) of the three developmental germ layers revealed that the teratomas formed by both cell lines had normal structure. These analyses indicated that the “artificial patient” cell line (AP) maintained a pluripotent state. Specificity, i.e., the absence of undesired mutations is essential for any gene-editing approach. To test the specificity of cleavage by TALEN, we used e-PCR to examine potential off-target sites in the human genome. We selected ten genomic sites, which were homologous to the designed TALEN recognition sequence (Table 1). We designed primers for all 10 potential off-target sites then amplified these regions from the genomic DNA extracted from the “artificial patient” iPSCs. DNA sequencing analysis revealed the absence of mutations at these predicted off-target sites. These data suggest that TALENs have high specificity for their targeting sequences.

### Differentiation of the Nor iPSCs into neurons after gene manipulation

**Figure 4:**
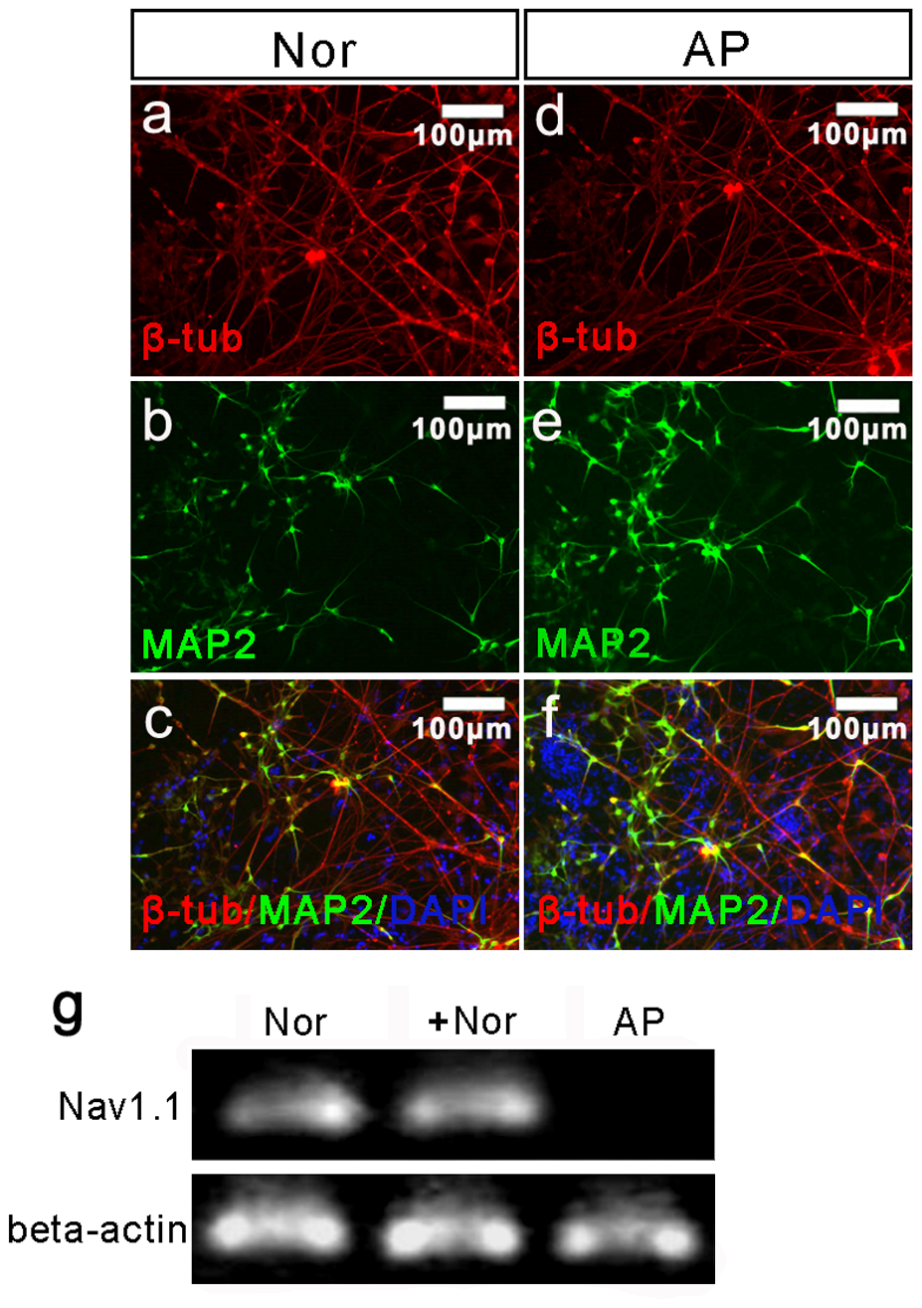
Characterization of differentiated neurons Immunofluorescence staining of the neuron-specific markers β-tubulin (red) and microtubule-associated protein 2 (green) in the neurons differentiated from the “Nor” (a/b/c) and the “AP” (d/e/f) cells. The scale is 100 μm. (g) RT-PCR analysis of the neurons indicated that gene manipulation did not disrupt expression of the Nav1.1 channel. “NC” indicates the negative control iPSCs. “Beta-actin” was used as the positive control (PC). “Nor” indicates the neurons differentiated from normal iPSCs, and “AP” indicates the “artificial patient” neurons obtained after gene manipulation. The full-length gel is shown in Supplemental Figure S5.

**Figure 5:**
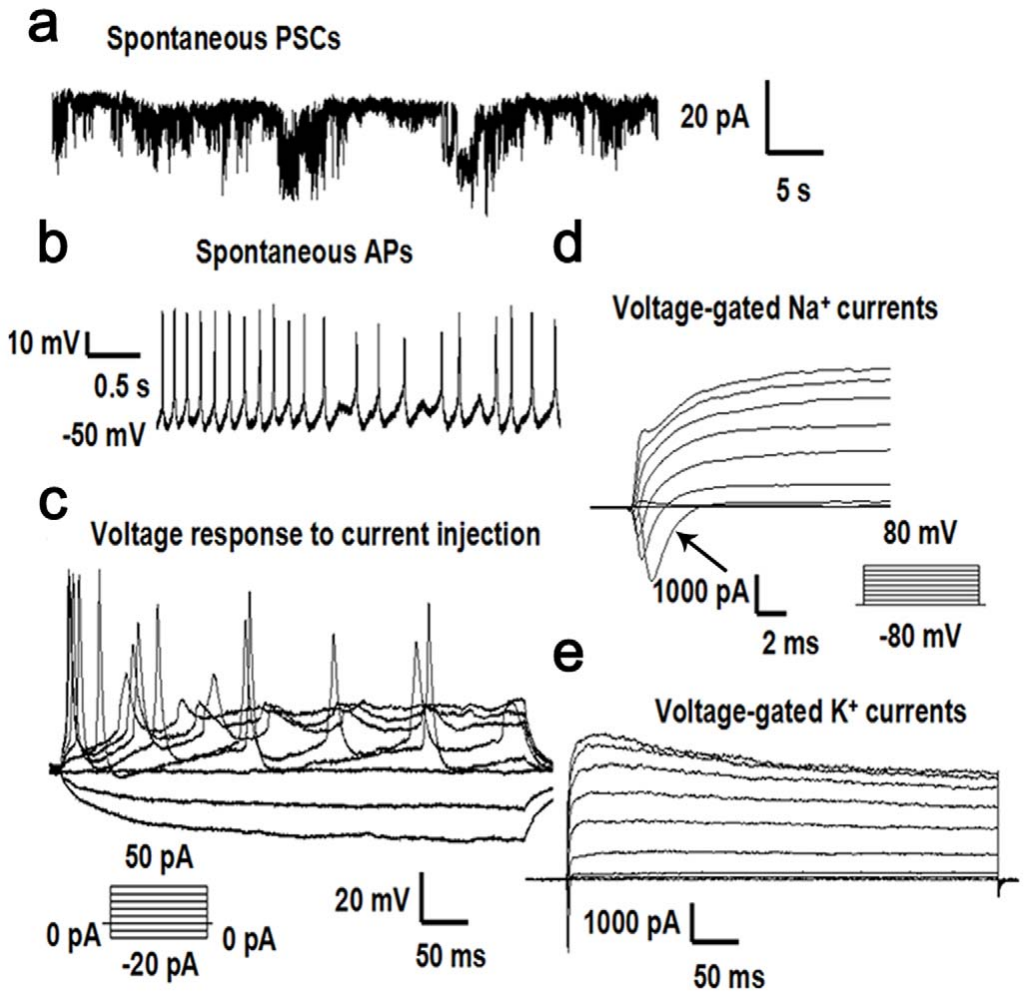
Electrophysiological characteristics of neurons differentiated from normal iPSCs (a) shows a whole-cell neuronal recording in which spontaneous miniature excitatory post-synaptic currents (PSCs) were detected. (b) shows the spontaneous action potentials (APs) without current injection. (c) indicates the evoked action potentials recorded after injection of step currents (−20 to 50 pA). (d) shows the voltage-gated sodium currents, indicated by the arrow, recorded following depolarizing voltage steps (−80 to 80 mV). (e) indicates the voltage-gated potassium currents evoked following depolarizing voltage steps (−80 to 80 mV).

**Figure 6:**
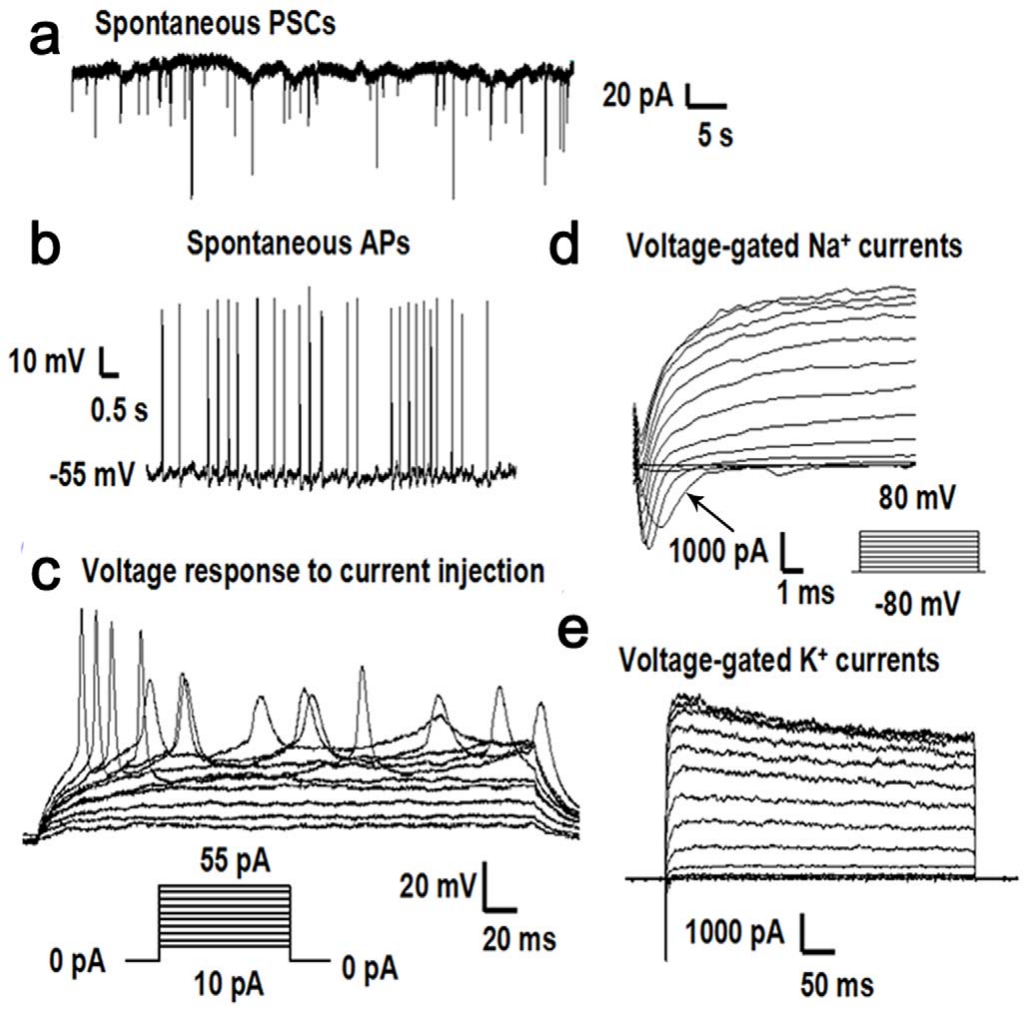
Electrophysiological characteristics of neurons differentiated from “artificial patient” iPSCs (a) shows a whole-cell neuronal recording in which spontaneous miniature excitatory post-synaptic currents (PSCs) were detected. (b) shows spontaneous action potentials (APs) without current injection. (c) indicates the evoked action potentials recorded after injection of step currents (−20 to 50 pA). (d) shows the voltage-gated sodium currents, indicated by the arrow, recorded following depolarizing voltage steps (−80 to 80 mV). (e) indicates the voltage-gated potassium currents evoked following depolarizing voltage steps (−80 to 80 mV).

Using the TALEN technology, we introduced the A5768G mutation in the “Nor” cell line. To verify that this genetic manipulation did not disrupt expression of the Nav1.1 α subunit, we used an embryoid body (EB)-based protocol to induce neural differentiation of the parental (Nor) and targeted (AP) iPSC lines as described under Methods. Immunofluorescence staining analysis indicated that the neuron-specific markers TuJ1 and Map2 were expressed in the neurons derived from the parental and targeted iPSCs lines (Figure 4a/b/c/d/e/f). After Cre-mediated excision of the drug-resistance cassette, RT-PCR analysis was performed using Nav1.1 α subunit-specific primers; this analysis confirmed that the gene manipulation did not affect expression of the Nav1.1 α subunit in the targeted cell line (Figure 4g). To further characterize the neurons, we performed electrophysiological recordings using the neurons derived from the parental “Nor” cell line and the “AP” cell line respectively. Analysis of patch-clamp recordings revealed that the neurons formed functional synapses with miniature excitatory post-synaptic currents (PSC) (Figure 5a). The neurons had spontaneous action potentials and evoked action potentials after injection step currents (Figure 5b,c). Furthermore, the neurons exhibited sodium and potassium channel activities by generating inward voltage-gated sodium currents and outward voltage-gated potassium currents (Figure 5d, e). Analysis of the “artificial patients” cell line (Figure 6) revealed similar neuronal characteristics as well as sodium and potassium channel activity compared to the normal cell line, indicating successful differentiation into functional neurons. Furthermore, the differentiated neurons could form an integrated neuronal circuit in vitro.

## Discussion

Several approaches such as transgenic animal models and cultured human cells are used to study the pathological mechanism of the neurodegenerative disease SMEI. Due to the lack of patient-derived, disease-relevant cell types and tissues, these approaches only partially recapitulate the molecular and cellular changes observed in patients. The development of iPSCs provides a novel approach to solve this problem. A crucial limitation in the use of iPSC technology is the inability to perform experiments under genetically defined conditions. Gene targeting mediated by traditional homologous recombination in hiPSC is generally difficult and inefficient. In this study, we generated TALENs with highly specific cleavage activity in human iPS cells. Using the TALEN technology, we introduced the epilepsy-related A5768G mutation into normal hiPSC. The resulting “artificial patient” iPS cell lines remained stably pluripotent and could successfully differentiate into neurons with normal functional characteristics. Furthermore, the TALENs did not generate off-target mutations, which is essential for their further application. Therefore, the TALEN technology is an efficient and effective method for gene manipulation to introduce disease-causing mutations in human pluripotent stem cells.

Use of the TALEN technology reduces the time required for plasmid construction. Based on our previous studies, we reduced the duration of the experimental scheme to approximately 2–3 weeks with subsequent drug screening. HiPSCs have poor survival, especially after electroporation. To promote survival, the cells were plated together on 12-well or 6-well dishes. After growth of the drug-resistant clones, the colonies were digested to obtain single cells, which grew into single-cell clones after approximately 2 weeks. While in our project, we plated the cells directly on 96-well dishes and ensured one cell per well survived after electroporation. We optimized conditions to promote survival. First, the “used” cell media were collected before electroporation on the third and the fourth day after passage when the clone coverage reached 40%–70%. Then, the dead cells were removed by centrifugation, and the supernatant was filtered with a 0.22-μm filter membrane. We mixed the supernatant and the fresh medium (mTesR) in the ratio of 1:3–1:5 to obtain an “adaptive medium”. The “adaptive medium” and an apoptosis-inhibitor ((ROCK)-inhibitor, Y27632) were used for the first 7 days after electroporation. The surviving clones were screened by G418 selection for the next 6–10 days until they could be passaged. During this period, the concentration of the drug was increased gradually to 100 µg/ml day by day. Then, the genomic DNA was extracted for subsequent analysis. This process is easy and efficient.

This method significantly eliminated genetic background noise. This facilitated the accurate discrimination of disease-relevant effects. However, the pathogenesis of SMEI is very complicated. Different pathological mechanisms might underlie a common epileptic phenotype. The hyperexcitable state of neuronal network, which causes seizures in SMEI, is considered to occur by alteration of the normal balance of excitatory glutamatergic neurons and inhibitory GABAergic neurons. In this study, mixed neurons, including glutamatergic and GABAergic neurons, were obtained by differentiation. The neurons were recorded for preliminary identification. The electrophysiological phenotypes associated with disease are more complicated and require further studies including the distinction of different types of neurons. Several methods are used to study pathological mechanisms, such as marking the cells used for patch-clamp recording with biocytin and subsequent characterization by immunohistochemical analysis^18^. The establishment of disease models represents a significant advance for basic biomedical research and a platform for large-scale drug screening. Mouse models have facilitated the generation of disease-specific genetically corrected iPSC-derived cells for disease treatment, paving the way for further studies^19^. However, further studies are required to realize the potential of iPSC technology for hiPSC-based cell replacement therapies.

## Methods

### Cell culture and construction of TALENs and the donor vector

Normal iPSCs (Nor) were cultured on MG (Sigma, Cat. No. 354277) coated 6-well plates with mTESR medium (Sigma, Cat. No.05850). The cells were passaged every 4 to 6 days with dispase (0.001 g/ml, Sigma). The SCN1A TALENs were constructed as described previously^20^. The donor vector was constructed as described in the text.

### Efficiency of TALENs

To test the cleavage activity of the designed TALENs, normal iPSCs (Nor) were transfected with two TALEN plasmids by electroporation. Cells were planted on MG-coated 6-well plates containing mTESR medium for 3–4 days. The genomic DNA of the cells was extracted. The targeted region was amplified by PCR using the primers Fs/Rs (Supplemental Table S1), cloned into the pMD-18T vector (TaKaRa, Cat. 6011), and sequenced to examine the indel mutation frequency.

### Homologous recombination-based SCN1A targeting in hiPSCs using TALENs

hiPSCs (Nor) were cultured in Rho Kinase (ROCK)-inhibitor (Sigma, Y-27632, Cat.No.Y0503) 24 hours prior to electroporation. Cells were harvested using Accutase (Sigma, Cat. No. A6964-100 ML) for 7–15 min, and 8 × 10^5^ cells were resuspended in 100 μl Nucleofector® Solution (Lonza) at room temperature. A total of 1–5 µg of donor plasmids and 5–10 µg of each TALEN-encoding plasmid were used to electroporate cells using Human Stem Cell Nucleofector® Kit 2 (Lonza, Cat. No.VPH-5022). For electroporation, we used the A23 procedure in the Amaxa Nucleofector 11 Device (Lonza). The cuvettes in the Human Stem Cell Nucleofector® Kit 2 were used. Single cells were subsequently plated on matrigel-coated 96-well plates containing mTESR medium supplemented with ROCK-inhibitor for the first 7 days and single-cell colonies were expanded after G418 selection (100 µg/ml) for 13 to 17 days. The genomic DNA was extracted using the TIANamp Genomic DNA Kit (Tiangen, Cat. No. DP304-02). Approximately 200 ng of DNA was used as input for each PCR using the F0/R0 primers and the enzyme KOD-Plus-Neo (ToYoBo, Code: KOD-401). The PCR products were sequenced to verify the disease-related point mutation.

### Removal of the Neo cassette by transient expression of the Cre recombinase

The gene-manipulated Nor (+Nor) iPSCs (8 × 10^5^) cells were electroporated with plasmids expressing the Cre recombinase (10 µg) as described previously. The single-cell colonies were selected 10 to 14 days after electroporation for extraction of genomic DNA. Excision of the Neo cassette was verified by sequencing the PCR products using the R1′/F2′ primers.

### Immunocytochemistry

Cells were fixed in 4% paraformaldehyde in PBS and immunostained using standard protocols. Antibodies against Nanog (Rabbit mAb, Cat.No. 4903s, Cell Signaling) and TRA-1-81 (Mouse mAb, No. 4745s, Cell Signaling) were used to verify the pluripotency of the iPSCs after gene editing. The antibodies used to identify the neurons derived from the iPSCs were neuron-specific class III β-tubulin (TuJ, Rabbit mAb, Cat.AB15708, Millipore) and Microtubule-associated protein 2 (Map 2, Mouse mAb, Cat. AB34918, Millipore). The cells were incubated at 4°C overnight in the primary antibodies, which were diluted with 10% goat serum and 0.3% Triton X-100 in PBS. The dilutions used were 1:800 (Nanog), 1:1000 (TRA-1-81), 1:400 (Map), and 1:200 (TuJ1). The cells were then treated with a secondary antibody (goat-anti mouse Alexa 555, 1:200, Cell Signaling Technology, Cat. #4409 and/or Alexa Fluor® 594 goat-anti rabbit Alexa 488, 1:200, Cell Signaling Technology, Cat. #4412) diluted in the same solution with the primary antibodies for 60 minutes at room temperature. The cells were then stained with a DAPI solution (diluted in PBS with the rate 1:1000) for 10 minutes. Between these steps, the cells were rinsed three times with 0.5% Tween in PBS.

### Teratoma analysis

The iPSCs were digested into single cells by Accutase (7–15 min), collected by centrifugation, resuspended in 200 µl MG (2 × 10^6^ cells), and injected subcutaneously into the right hind leg of immunocompromised NOD-SCID mice (SLAC LABORATORY ANIMAL, Shanghai, China). Tumors generally developed within 8 weeks, and animals were sacrificed before the tumor size exceeded 1.5 cm in diameter. Teratomas were isolated after sacrificing the mice, fixed in formalin, and embedded in paraffin. After sectioning, the teratomas were diagnosed based on hematoxylin and eosin (HE) staining and were observed with the LEICA DMI6000B (Leica Microsystems GmbH) inverted fluorescence microscope.

### Neuronal differentiation

The iPSCs were differentiated to neurons as described previously^21^. This method of neuronal conversion involves an embryoid body step. Normal iPSCs were passaged using dispase (Sigma), and cell clumps were cultured on feeder cells (Cyagen, Cat. No.MUIEF-01002), which were seeded one day earlier in EB culture medium containing F12/DMEM (life technology) supplemented with 20% KSR (Life Technologies), 1% glutinmax (life technology), 1% NEAA (life technology), 0.1% β-mercaptoethanol (life technology), 5 μM TGF-β RI Kinase Inhibitor VI, and 5 μM Dorsomorphin. This time point was defined as day zero of differentiation. On the fourth day, the medium was changed to N2 medium containing F12/DMEM supplemented with 1% N2 (life technology), 1% NEAA (life technology); 2 μg/ml heparin. On the sixth day, the EBs were collected and plated on MG-coated plates in N2 medium. The neuronal rosettes appeared 10–12 days after incubation. On the sixteenth day, the rosettes were picked and plated on MG-coated plates in N2 medium. On the seventeenth day, the N2 medium was replaced with neuron differentiation medium containing F12/DMEM supplemented with 1% N2 (life technology), 2% B27 (life technology), 1 ng/ml BDNF (Peprotech), 1 ng/ml GDNF (peprotech), and 1 μmol cAMP (Sigma). The cells were cultured in neuron differentiation medium for more than 2 weeks to obtain full maturation.

### Electrophysiology

Electrophysiological experiments were performed on mature neurons derived from normal iPSCs. Whole-cell patch-clamp recordings were performed in either voltage or current clamp mode to measure voltage-activated sodium/potassium currents and action potentials. An Axopatch 200B amplifier (Axon Instruments, USA) was used to record the electrophysiological signals. The data were acquired and analyzed using Clampfit 10.2 software (Molecular Devices, USA). Borosilicate glass pipettes had resistances of 4–8 MΩ when filled with a solution containing the following (mM): 140 potassium methanesulfonate, 10 HEPES, 5 NaCl, 1 CaCl_2_, 0.2 EGTA, 3 ATP-Na_2_, 0.4 GTP-Na_2_, pH 7.2 (adjusted with KOH). The bath solution contained the following (mM): 127 NaCl, 3 KCl, 1 MgSO_4_, 26 NaHCO_3_, 1.25 NaH_2_PO_4_, 10 D-glucose, 2CaCl_2_, pH 7.4 (adjusted with NaOH). Cells plated on coverslips were placed in a submerged recording chamber and were continually perfused with the bath solution equilibrated with 95% O_2_ and 5% CO_2_. All electrophysiological experiments were performed at room temperature.

### RT-PCR analysis

On the 36th day after differentiation, RNA was extracted from the neurons. The RNA was reverse transcribed to cDNA using the Prime Script^TM^ RT reagent Kit (TAKARA, Cat.DRR037S). PCR was then performed using Nav1.1 α subunit specific primers.

### TALEN off-target analysis

To identify potential off-target sites of *SCN1A* TALENs, we used e-PCR (www.ncbi.nlm.nih.gov/sutils/e-pcr) to scan the human genomic sequence. The criteria for determining off-target sites included up to 2 bp of mismatches, 2 bp of gaps, and <300 bp of spacer sequence between the two putative binding sites. The identified potential off-target regions were amplified by PCR using genomic DNA from “artificial patient” iPSCs as template and sequenced to detect any off-target effects (The sequences of primers F1/R1, F2/R2, F4/R4, F5/R5, F6/R6, F7/R7, F8/R8, F9/R9, F10/R10 are listed in Supplemental Table S1).

### Ethics statement

The Guangzhou Institute of Biomedicine and Health, Chinese Academy of Sciences and Ethics committee had approved the experiments, including any relevant details. All experiments were performed in accordance with relevant guidelines and regulations.

## Author Contributions

W.C., J.L., X.G. contributed to design the study; W.C. and Z.L. wrote the main manuscript text; W.C., X.G. and L.Z. prepared Figures 1–6. All authors reviewed the manuscript.

## Supplementary Material

Supplementary Information Generation of the SCN1A epilepsy mutation in hiPS cells using the TALEN technique

## Figures and Tables

**Table 1:** Potential TALEN off-target sites identified by e-PCR analysis of the *Homo sapiens* genome. Ten potential off-target sites were identified. All sites were amplified and sequenced. Site No. 3 is the target site used in this study

No.	Chr	Strand	Strand from X to Y	Gene	Mism	Gaps	act_len/exp_len
1	1	+	21939118-21939256	RAR1GAP	2	2	139/16-300
2	1	-	45267881-45268081	PLK3	2	2	201/16-300
3	2	-	166847491-166847538	SCN1A	2	2	48/16-300
4	7	-	46889460-46889488	-	2	2	29/16-300
5	8	-	73538799-73539044	KCNB2	2	2	246/16-300
6	8	-	136252292-136252487	RP11-452N4.1	2	2	196/16-300
7	13	+	67903014-67903175	-	2	2	162/16-300
8	16	-	70839608-70839644	-	2	2	37/16-300
9	19	+	42535231-42535473	GRIK5	2	2	243/16-300
10	X	+	11051438-11051553	-	2	2	116/16-300
