# Supplementary material for: Generation of the SCN1A epilepsy mutation in hiPS cells using the TALEN technique
Source: Sci Rep. 2014 Jun 23;4:5404. doi: 10.1038/srep05404 (PMC4066246; doi:10.1038/srep05404)
Supplement: Supplementary Information — Generation of the SCN1A epilepsy mutation in hiPS cells using the TALEN technique [file srep05404-s1.pdf]

Supplemental Information

Generation of the SCN1A epilepsy mutation in hiPS cells using the TALEN technique

Wanjuan Chen[1#], Jingxin Liu[2#], Longmei Zhang[2], Huijuan Xu[3], Xiaogang Guo[3], Sihao Deng[4], Lipeng Liu[5], Daiguan Yu[3], Yonglong Chen[3] & Zhiyuan Li*[3,4]

[1]The School of Life Sciences, Anhui University, Hefei 230027; [2]The School of Life Sciences, University of Science and Technology of China, Hefei 230027; [3]Key Laboratory of Regenerative Biology, South China Institute for Stem Cell Biology and Regenerative Medicine, Guangzhou Institutes of Biomedicine and Health, Chinese Academy of Sciences, Guangzhou 510530, China; [4]Xiang-Ya School of Medicine, Central South University and [5]Xiang-Ya Boai Hospital, Changsha 410013, China.

Supplementary Table S1 The primers used in this study

Primer	Sequence
Fs	TAAGGTCAGTGCCTACAA
Rs	CCTACCCACAAATAATCTAC
F0	TACCCTGTTCCGAGTGATCCGTCTT
R0	CCCGGTCATAGGAAGGTGGACAA
R1'	CTGTTCTCACTACCAGCTGACACGAATTCAAA
F2'	CCATTGTGCATCTTATCTTCAGCAGGATCCAAAA
F1	GGAGTCTCGCTCTGTC
R1	TAGCAACGAACCCATA
F2	CGGAGCAGAGGAAGAA
R2	TCACCAAAGGCCAGAG
F4	AAAATATCTGCATTGGGACT
R4	TGTGGGTGGGTGACTGG
F5	GAGCCTGTACTGTATTT
R5	GTTAGCCTGGTTATGT
F6	AGGCAAAGATGGTCAC
R6	CAAGCCAGCACAAGAA
F7	TAGGAAATGCGGAAAG
R7	TCACCTGGCAGTTGTT
F8	AGCCTTTCATTACTTTC
R8	CTGATAACGGGTTCCT

F9	GGCTCCTTCTCAGCATTG
R9	ACTTCATTTGGGACAGCA
F10	TCCCACTCATCTTCCA
R10	ACCAGCCTTACCAACA

Supplementary Table S2 The sequencing at the cleavage site of TALENs to verify TALEN's efficiency

TALEN Binding Site	number
CACTACCAGCTGACACTGCTGAAGATAAGATGCACAATGGCTAGTCAG	The original sequence
CACTACCAGCTGACACTGCTGAAGATAAGATGCACAATGGCTAGTCAG	x26
CACTACCAGCTGACACTGCCGAAGATAAGATGCACAATGGCTAGTCAG	x1
CACTACCAGCTGACACTGCTGAAGATATGATGCACAATGGCTAGTCAG	x1

Supplementary Figure S1 The DNA sequences of TALENs used in this study TALEN-L

GGATCTGCCACCATGGCTCCAAGAAGAAGCGTAAGGTAGACTACAAAGACCATGACGGTGATTATAAAGATCATGA
CATCGATTACAAGGATGACGATGACAAGGGTACCGTGGATCTACGCACGCTCGGCTACAGCCAGCAGCAACAGGAG
AAGATCAAACCGAAGGTTCGTTCGACAGTGGCGCAGCACCACGAGGCACTGGTCGGCCATGGGTTTACACACGCGC
ACATCGTTGCGCTCAGCCAACACCCGGCAGCGTTAGGGACCGTCGCTGTCAAGTATCAGGACATGATCGCAGCGTT
GCCAGAGGCGACACACGAAGCGATCGTTGGCGTCGGCAAACAGTGGTCCGGCGCACGCGCTCTGGAGGCCTTGCTC
ACGGTGGCGGGAGAGTTGAGAGGTCCACCGTTACAGTTGGACACAGGCCAACTTCTCAAGATTGCAAAACGTGGCG
GCGTGACCGCAGTGGAGGCAGTGCATGCATGGCGCAATGCACTGACGGGTGCCCCCCTGAACCTGACCCCGGACCA
AGTGGTGGCTATCGCCAGCCACGATGGCGGCAAGCAAGCGCTCGAAACGGTGCAGCGGCTGTTGCCGGTGCTGTGC
AGGACCATGGCCTGACCCCGGACCAAGTGGTGGCTATCGCCAGCAACATTGGCGGCAAGCAAGCGCTCGAAACGGT
GCAGCGGCTGTTGCCGGTGCTGTGCCAGGACCATGGCCTGACTCCGGACCAAGTGGTGGCTATCGCCAGCCACGAT
GGCGGCAAGCAAGCGCTCGAAACGGTGCAGCGGCTGTTGCCGGTGCTGTGCCAGGACCATGGCCTGACCCCGGACC
AAGTGGTGGCTATCGCCAGCAACGGTGGCGGCAAGCAAGCGCTCGAAACGGTGCAGCGGCTGTTGCCGGTGCTGTG
CCAGGACCATGGcCTGACCCCG

TALEN-R

GGATCTGCCACCATGGCTCCAAAGAAGAAGCGTAAGGTAGACTACAAAGACCATGACGGTGATTATAAAGATCATG
ACATCGATTACAAGGATGACGATGACAAGGGTACCGTGGATCTACGCACGCTCGGCTACAGCCAGCAGCAACAGGA
GAAGATCAAACCGAAGGTTCGTTCGACAGTGGCGCAGCACCACGAGGCACTGGTCGGCCATGGGTTTACACACGCG
CACATCGTTGCGCTCAGCCAACACCCGGCAGCGTTAGGGACCGTCGCTGTCAAGTATCAGGACATGATCGCAGCGT
TGCCAGAGGCGACACACGAAGCGATCGTTGGCGTCGGCAAACAGTGGTCCGGCGCACGCGCTCTGGAGGCCTTGCT
CACGGTGGCGGGAGAGTTGAGAGGTCCACCGTTACAGTTGGACACAGGCCAACTTCTCAAGATTGCAAAACGTGGC
GGCGTGACCGCAGTGGAGGCAGTGCATGCATGGCGCAATGCACTGACGGGTGCCCCCCTGAACCTGACCCCGGACC

AAGTGGTGGCTATCGCCAGCCACGATGGCGGCAAGCAAGCGCTCGAAACGGTGCAGCGGCTGTTGCCGGTGCTGTG
CCAGGACCATGGCCTGACCCCGGACCAAGTGGTGGCTATCGCCAGCAACGGTGGCGGCAAGCAAGCGCTCGAAACG
GTGCAGCGGCTGTTGCCGGTGCTGTGCCAGGACCATGGCCTGACCCCGGACCAAGTGGTGGCTATCGCCAGCAACA
ATGGCGGCAAGCAAGCGCTCGAAACGGTGCAGCGGCTGTTGCCGGTGCTGTGCCAGGACCATGGCCTGACCCCGGA
CCAAGTGGTGGCTATCGCCAGCAACATTGGCGGCAAGCAAGCGCTCGAAACGGTGCAGCGGCTGTTGCCGGTGCTG
TGCCAGGACCATGGGCCTGACTCCGGACCAAGTGGTG

Supplementary Figure S2 The donor plasmid’s sequence

GCTTTGTGGAAGGCGATCGGTGCGGGCCTCTTCGCTATTACGCCAGCTGGCGAAAGGGGGATGTGCTGCAAGGCGA
TTAAGTTGGGTAACGCCAGGGTTTTCCCAGTCACGACGTTGTAAAACGACGGCCAGTGAATTGTAATACGACTCAC
TATAGGGCGAATTGGAGCTCCACCGCGGTGGCGGCCGCGTATAGGTTAAGCAGTGTGTTTTTTTAAACATGTCTGT
ACAGTCTGGCTATATACCATATGTTATCCACTTAAAATGTAAAAATAACCAAAAAGCTGTTAAAGTGCTGCAAACT
ATTGCTTAATGACTTAAATAAATGAGATCTGTTGAACAATTTCCTTGACTTTACCACTGACATATGGTTTCTCATA
AATGAGATTCTGAGCAGTGAGAGAAACCAGATACAGCAGCATGGTAATATAAACATGCATTGATAGCATCCAAACT
ATCTATAAATGGTACAGAATACATTTTATTACCTGTGTAAAGCTTGCACTCTACATTTCTTGTGGTACATATTTGA
TGCAATAAATACTGTGCTTAGGTCATTATTTGTTTGCTCAAACGTGCACCACAGGGTAAAATGACTATTTACATAA
TAAATAGCATTTCATTAACATATACAGTGTCAACCTTGCTGAGAGCCGAAGATGGCTAAACAAAGTGCAGGAAAAA
GCAGAAATTTATAAAGAGTAATTTTGGTCAATTCAGTCTTCTGGCGGTGGAGGGTGAGGGGCAATATTCACTATTC
AGGTTTTTTTTTTAATACAACAAAAAGAAACATAACATTTATGACTCCAAACATTTGAATGAAGTTTGCACCTGCT
AAGATTTACTGGCCTACCCACAAACAATCTACTTGGTCTAGGGGCTGGATTTCGCAAAACAAGATCAACAAAGCAC
CTCCACTTATCACCCAATTACCCCTCCCAGAAATATGGTGAAATTTAAGAATTTGTTTTTCTTGTGACTTTTTCTC
ATGCATGATCTCTAAGTGCAGCATGCCCTCATGCAAACCACGACTTTGTGTAGCTGGGAGGGCCATGTGGTTGCCA
TACCCCCCAGGTGGCATACCTGTTATAGAGGTCCTTAGCCTATTTCTCAACAGAAACTTCGTTTACAAAAATAGTC
ACATATAATAAACACATGGATTAACAAAAAGATGAATCCACTAACAGATTCCATAAAAATGTGACAAATGTGGCAG
TTGAAATGCAAACAGTGGATACAATTACTACACTAAAGTGTTTCATGTTAAACAACCCCAAAATCACAGGTTTGCA
CCCCTTGAAACTGGTCCCTACAGTCTGACTAGCCATTGTGCATCTTATCTTCAGCAGGATCCCCTCGAGGGACCTA
ATAACTTCGTATAGCATACATTATACGAAGTTATATTAAGGGTTCCGCAAGCTCTAGTCGAGCCCCAGCTGGTTCT
TTCCGCCTCAGAAGCCATAGAGCCCACCGCATCCCCAGCATGCCTGCTATTGTCTTCCCAATCCTCCCCCTTGCTG
TCCTGCCCCACCCCACCCCCCAGAATAGAATGACACCTACTCAGACAATGCGATGCAATTTCCTCATTTTATTAGG
AAAGGACAGTGGGAGTGGCACCTTCCAGGGTCAAGGAAGGCACGGGGGAGGGGCAAACAACAGATGGCTGGCAACT
AGAAGGCACAGTCGAGGCTGATCAGCGAGCTCTAGAGAATTGATCCCCTCAGAAGAACTCGTCAAGAAGGCGATAG
AAGGCGATGCGCTGCGAATCGGGAGCGGCGATACCGTAAAGCACGAGGAAGCGGTCAGCCCATTCGCCGCCAAGCT
CTTCAGCAATATCACGGGTAGCCAACGCTATGTCCTGATAGCGGTCCGCCACACCCAGCCGGCCACAGTCGATGAA
TCCAGAAAAGCGGCCATTTTCCACCATGATATTCGGCAAGCAGGCATCGCCATGGGTCACGACGAGATCATCGCCG
TCGGGCATGCGCGCCTTGAGCCTGGCGAACAGTTCGGCTGGCGCGAGCCCCTGATGCTCTTCGTCCAGATCATCCT
GATCGACAAGACCGGCTTCCATCCGAGTACGTGCTCGCTCGATGCGATGTTTCGCTTGGTGGTCGAATGGGCAGGT
AGCCGGATCAAGCGTATGCAGCCGCCGCATTGCATCAGCCATGATGGATACTTTCTCGGCAGGAGCAAGGTGAGAT
GACAGGAGATCCTGCCCCGGCACTTCGCCCAATAGCAGCCAGTCCCTTCCCGCTTCAGTGACAACGTCGAGCACAG
CTGCGCAAGGAACGCCCGTCGTGGCCAGCCACGATAGCCGCGCTGCCTCGTCCTGCAGTTCATTCAGGGCACCGGA
CAGGTCGGTCTTGACAAAAAGAACCGGGCGCCCCTGCGCTGACAGCCGGAACACGGCGGCATCAGAGCAGCCGATT
GTCTGTTGTGCCCAGTCATAGCCGAATAGCCTCTCCACCCAAGCGGCCGGAGAACCTGCGTGCAATCCATCTTGTT
CAATGGCCGATCCCATGGTTTAGTTCCTCACCTTGTCGTATTATACTATGCCGATATACTATGCCGATGATTAATT
GTCAACAGGCTGCAGGTCGAAAGGCCCGGAGATGAGGAAGAGGAGAACAGCGCGGCAGACGTGCGCTTTTGAAGCG
TGCAGAATGCCGGGCCTCCGGAGGACCTTCGGGCGCCCGCCCCGCCCCTGAGCCCGCCCCTGAGCCCGCCCCCGGA

CCCACCCCTTCCCAGCCTCTGAGCCCAGAAAGCGAAGGAGCAAAGCTGCTATTGGCCGCTGCCCCAAAGGCCTACC
CGCTTCCATTGCTCAGCGGTGCTGTCCATCTGCACGAGACTAGTGAGACGTGCTACTTCCATTTGTCACGTCCTGC
ACGACGCGAGCTGCGGGGCGGGGGGGAACTTCCTGACTAGGGGAGGAGTAGAAGGTGGCGCGAAGGGGCCACCAAA
GAACGGAGCCGGTTGGCGCCTACCGGTGGATGTGGAATGTGTGCGAGGCCAGAGGCCACTTGTGTAGCGCCAAGTG
CCCAGCGGGGCTGCTAAAGCGCATGCTCCAGACTGCCTTGGGAAAAGCGCCTCCCCTACCCGGTAGAATTTCGACG
ACCTGCAGCCAAGCTAGCTTGGCTGGACGTAAACTCCTCTTCAGACCTAATAACTTCGTATAGCATACATTATACG
AAGTTATATTAAGGGTTATTGAATATGATCGGAATTGGGCTGCAGGAATTCGTGTCAGCTGGTAGTGAGAACAGTA
ACCTCCTGTCAAGGTCATCTCCCCTTTACACAGAGTCACAGTTTGCTGACAAGGGGTCACTGTCTTATTGTAGGCA
CTGACCTTAAGGAGATTTGTGTAAAAACAGTCAGTTTGGCATTGACCTCCTAAAGGAGTCCTGTTGATAAAAATAC
ATCACCTTCACAGGCTGTAAACAATTTGTCACCCAATTATTTTTATTTATTTTCATTTATTTCCCTTTGGCTTTTT
CATCTTTGCCTTCTTGCTCATGTTTTTCCACAATTGGCTTTGTCACCCGGTCATAGGAAGGTGGACAAGCTGCAGT
GGACATGGTCAGATCAGTTTTTTCTGTAATAGAGTTTCATTTATTCTGTCAATTATCATGTCTTCTTTTATAAGAA
GATTAGCCCCACCTTTGATTTTGTTTTTATTGTACGTAAAGGAAGCTTGTTTTACAGTTCGCTTTAAAAGGTGGCG
TCTGTAAGCACGCTGAATAATGACAGCAGATACTTCCTCTTGTTTTCGTTTTAAAGTAGTAGTGATTGGCTGATAG
GAGACCTTGGAAGGATTGGAAGCCATGAATCGCTCTTCCATCTGTATTCGTAGAGCATCCATCTCTCCACTCTCTC
CTAGAACCCGCTTTGTAAAAGCAAATAAGATATCAAGACAGTGGATCCGGTCACCACTCACCATGGGCAAATCCAT
GGCAATGAGCTGGAGTTTGTTTGGTTGTGGCAGATTGAGAGGCGGTTCAAGCGCAGCTGCAAACTGAGATAATTTT
TCAAATTCCATGAACTGAGTTGCATCGGGATCAAACTTCTCCCAAACCTCATAGAACATCTCAAAGTCATCCTCAC
TCAGAGGCTCTGCACTTTCTTCAGTAGCAACACTGAAGTTCTCCAGGATGACCGCGATGTACATGTTCACCACAAC
CAGGAAGGATATGATGATGTAACTGACAAAAAAGAAAATTCCAACAGATGGGTTCCCACAGTCTCCCTTAACTGAG
CTTCCAGGGTTAACTTTATTAGGGTCACAGTCGGGTGGCTTACTGTTGAGAATGGGTGCTAGCAATCCATCCCAGC
CAGCAGAGGTTGTAATTTGGAATAGGCAGATCATGCTGTTGCCAAAGGTCTCAAAGTTGAACATGTCATCGATCCC
AACTTCCCTCTTAACATAGGCAAAGTTGGACATCCCAAAGATGGCGTAGATGAACATGACTAGGAAGAGTAGGAGG
CCGATGTTAAACAACGCAGGAAGGGACATCATCAAAGCAAAGGGTACCAGCTTTTGTCCCTTTAGTGAGGGTAATT
CGAGGCTGAAG

Supplementary Figure S3 The sequence of the normal iPSCs with the Neo (+Nor) used the primers R1’/F2’

ACGGGGCGAGACTGAATAACTTCGATAGCAGACATTATACGTAGTGAGTTAAGGGTCCGGAAGATCTAGTCGAGTT
TGGATTCGTGTTTCCTCCTCGGAAGAGTGAAGCCCACCGCATCCCCAGCATGCCTGCTATTGTCTTCCCAATCCTC
CCCCTTGCTGTCCTGCCCCACCCCACCCCCCAGAATAGAATGACACCTACTCAGACAATGCGATGCAATTTCCTCA
TTTTATTAGGAAAGGACAGTGGGAGTGGCACCTTCCAGGGTCAAGGAAGGCACGGGGGAGGGGCAAACAACAGATG
GCTGGCAACTAGAAGGCACAGTCGAGGCTGATCAGCGAGCTCTAGAGAATTGATCCCCTCAGAAGAACTCGTCAAG
AAGGCGATAGAAGGCGATGCGCTGCGAATCGGGGGCGGCGATACCGTAAAGCACGAGGAAGCGGTCAGCCCATTCG
CCGCCAAGCTCTTCAGCAATATCACGGGTAGCCAACGCTATGTCCTGATAGCGGTCCGCCACACCCAGCCGGCCAC
AGTCGATGAATCCAGAAAAGCGGCCATTTTCCACCATGATATTCGGCAAGCAGGCATCGCCATGGGTCACGACGAG
ATCATCGCCGTCGGGCATGCGCGCCTTGAGCCTGGCGAACAGTTCGGCTGGCGCGAGCCCCTGATGCTCTTCGTCC
AGATCATCCTGATCGACAAGACCGGCTTCCATCCGAGTACGTGCTCGCTCGATGCGATGTTTCGCTTGGTGGTCGA
ATGGGCAGATAGCCGGATCAAGCGTATGCAGCCGCCGCATTGCATCAGCAATGATGGATACTTTCCTCGTCAGGAG
CAAGGTGAGATGACAGCAGATCCTTCCCCCGTCACTTCGCCCAATAGCAGCCAGTCCCTTCACGCTTTCAGTGACA
GCGTCGAGCACAGCTGCGCAAGGAACGCACGTCGTGTCATGCCACGATAGCCGCCTGCCTCGTCCTGCAGTTCAAT
CAGGACACGGACAGTCGTCAGACAAAAGACGGGTGCTCTACTTGACATCGTAACACGAGAATCAGACTAGCGATGA
CTGTATGGCCAGCCATAGCGAATGCTGTCACTAGCGTGAGAACTAGCGCAATCACTTGTCAGTGCCGATCCATGTA
AGTCTCACTGTCGATACATGCTATAGCGGATTTACTTCACGTTCGATGCAAAGTGCA

Supplementary Figure S4 The full-length gel of Figure 2-d

It shows DNA gel electrophoresis of the PCR production of Neo cassette with the primers (R1'/F2') in the normal cell line (Nor), the normal gene targeted cell line (+Nor) and the "artificial patient" cell line (AP) after the removal of Neo cassette.

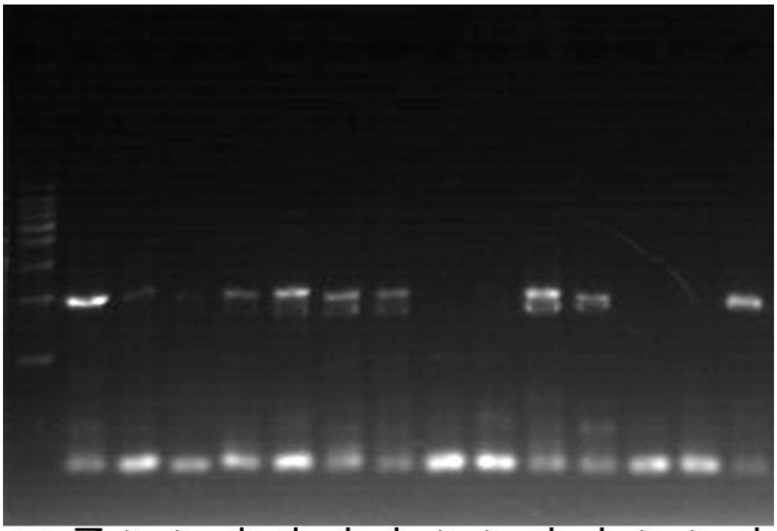

M Nor AP AP +Nor +Nor +Nor +Nor AP AP +Nor +Nor AP AP +Nor

Supplementary Figure S5 The full-length gel of Figure 4-g

The PT-PCR of the neurons indicated that the gene operation did not destruct the expression of Nav1.1 channel. The "NC" is the negative control which we used iPSCs. The "beta-actin" is the positive control (PC). The "Nor" is the neurons differentiated from the normal iPSCs and the "AP" indicates the "artificial patient" neurons after gene manipulation.

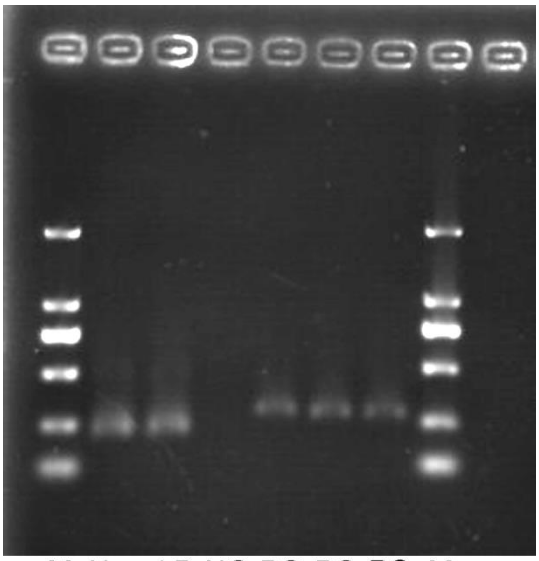

M Nor AP NC PC PC PC M
